# Evaluation of delivered dose to a moving target by 4D dose reconstruction in gated volumetric modulated arc therapy

**DOI:** 10.1371/journal.pone.0202765

**Published:** 2018-09-07

**Authors:** Hyekyun Chung, Jinhong Jung, Chiyoung Jeong, Jungwon Kwak, Jin-hong Park, Su Ssan Kim, Sang Min Yoon, Si Yeol Song, Jong Hoon Kim, Eun Kyung Choi, Seungryong Cho, Byungchul Cho

**Affiliations:** 1 Department of Radiation Oncology, Asan Medical Center, University of Ulsan, College of Medicine, Seoul, Korea; 2 Department of Nuclear and Quantum Engineering, Korea Advanced Institute of Science and Technology, Daejeon, Korea; North Shore Long Island Jewish Health System, UNITED STATES

## Abstract

**Purpose:**

To develop a 4D dose reconstruction method and to evaluate the delivered dose in respiratory-gated volumetric modulated arc therapy (VMAT).

**Materials and methods:**

A total 112 treatment sessions of gated VMAT for 30 stereotactic body radiotherapy (SBRT) patients (10 lung, 10 liver, and 10 pancreas) were evaluated. For respiratory-gated SBRT, 4DCT was acquired, and the CT data at the end-exhale phase was used for a VMAT plan. The delivered dose was reconstructed using a patient’s respiratory motion and machine motion acquired during the beam delivery. The machine motion was obtained from the treatment log file, while the target position was estimated from an external respiratory marker position. The target position was divided into 1-mm position bins, and sub-beams with beam isocenters corresponding to each position bin were created in a motion mimicking plan, reflecting motion data including MLC leaf positions and gantry angle and target position data during beam treatment. The reconstructed 4D dose was compared with the dose of the original plan using these dosimetric parameters; the maximum dose (D_max_) and mean dose (D_mean_) of gross target volume (GTV) or organs at risk (spinal cord, esophagus, heart, duodenum, kidney, spinal cord, and stomach). The minimum dose (D_min_) to GTV was also calculated to verify cold spots in tumors.

**Results:**

There was no significant difference of dose parameters regard to the GTV in all tumors. For the liver cases, there were significant differences in the D_max_ of duodenum (-4.2 ± 1.4%), stomach (-3.5 ± 4.2%), left kidney (-4.1 ± 2.8%), and right kidney (-3.2 ± 1.3%), and in the D_mean_ of duodenum (-3.8 ± 1.4%), stomach (-3.9 ± 2.2%), left kidney (-3.1 ± 2.8%), and right kidney (-4.1 ± 2.6%). For the pancreas cases, there were significant differences in the D_max_ of stomach (2.1 ± 3.0%), and in the D_mean_ of liver (1.5 ± 0.6%), duodenum (-1.0 ± 1.4%), stomach (2.1 ± 1.6%), and right kidney (-1.3 ± 0.9%). The average gamma pass rates were 97.6 ± 4.8% for lung cases, 99.6 ± 0.5% for liver cases, and 99.5 ± 0.5% for pancreas cases. Most cases showed insignificant dose variation, with gamma pass rates higher than 98%, except for two lung cases with gamma pass rates of 86.9% and 90.6%. The low gamma pass rates showed larger global motion ranges resulting from the baseline shift during beam delivery.

**Conclusion:**

The actual delivered dose in thoracic and abdominal VMAT under breathing motion was verified by 4D dose reconstruction using typical treatment equipment and software. The proposed method provides a verification method for the actual delivered dose and could be a dosimetric verification QA tool for radiation treatment under various respiratory management techniques.

## Introduction

Stereotactic body radiation therapy (SBRT) delivers ablative doses to tumors with stereotactic localization accuracy, resulting in promising local control [1–8]. Several studies showed potential benefit of a higher radiation dose with regard to local control, and subsequent overall survival [9–11]. However, many critical normal organs surrounding a tumor, especially gastrointestinal organs, are radiosensitive. Therefore, SBRT requires a high level of confidence in terms of the accuracy of the entire treament procedure, from simulation to beam delivery, in order to ensure safe and effective SBRT.

In recent years, SBRT volumetric modulated arc therapy (VMAT) has been widely used because of its short treatment time compared with conventional fixed field intensity-modulated radiation therapy (IMRT), while maintaining a high conformity of dose distribution in order to minimize irradiated dose to normal organs [12–14]. However, tumor and organ motion presents a challenge for SBRT to maintain high plan quality in accuracy and precision during beam delivery. Especially for thoracic and abdominal sites, respiratory motion could cause degradation in the highly conformal dose distribution expected in the planning stage, such as a blurring, shifting, averaging, and interplay effect between the tumor motion and movement of the beam delivery apparatus [15–17]. Several respiratory motion management strategies through four-dimensional computed tomography (4DCT) such as inclusive, gating, and tracking method, or 4D dose calculations with deformable image registration (DIR) are applied in SBRT [18–22]. However, the actual delivered dose during treatment, which included baseline shift, irregular respiratory amplitude, and a phase difference in respiratory motion, have not been confirmed. Therefore, 4D reconstruction of an actual delivered dose is required in order to ensure safe and effective SBRT, which make dose escalation of SBRT and subsequent improvement of local control possible.

Poulsen et al. [23] suggested a method for the actual 4D dose calculation that covers the irregularity of real breathing motion during beam delivery. They estimated the actual target position from kilovoltage intrafraction monitoring (KIM) [24] using a probability-based method that was developed to reconstruct the 3D target trajectory from 2D projection images [25]. The tumor and organ motion were rigidly modeled from the estimated target position and combined with the machine motion to generate a motion mimicking plan. Poulsen et al. applied their 4D dose calculation method to evaluate the delivered dose for six liver SBRT patients treated by VMAT [26]. However, the method is not widely used clinically because of the required in-house development of tumor position monitoring tools such as KIM.

In this study, we adapted the method of 4D delivered dose reconstruction for general clinical applications, such that it can be applied to more widely used clinical systems. For this purpose, we utilized an external surrogate signal that is widely used for gated beam delivery to estimate the tumor position. With this implementation, we evaluated the actual, delivered dose of gated VMAT SBRT for thoracic and abdominal cancer patients.

## Materials and methods

### Patient details and imaging

Thirty patients treated with SBRT were enrolled in this study. Three groups of ten patients each were lung, liver, and pancreas cancer patients. This study was approved by the Institutional Review Board of Asan Medical Center, and informed consent was waived because of the retrospective nature of the study. For each patient, 4DCT simulation was performed while the patient was breathing freely, in order to assess the respiratory-induced tumor motion. A multislice CT scanner (Lightspeed RT16, GE Healthcare Technologies, Waukesha, WI) was operated in the “axial cine mode,” where at each couch position, projection data were acquired continuously during multiple CT tube rotations over a full respiratory cycle (0.5 s rotation time, eight slices of 0.25 cm thickness). During 4DCT scanning and subsequent treatment, a Real-time Position Management (RPM) system (Varian Medical Systems, Palo Alto, CA) was used for respiratory motion monitoring. An external marker was located on the patient’s abdominal surface and was tracked by an optical camera in the RPM system. By looking-up the RPM signal recorded at the acquisition time of a CT image, each 4DCT image was sorted into 10 respiratory phases, from 0% (end-inhale) to 90% phases at intervals of 10%. The tumor motion in each direction (Left/Right, Anterior/Posterior, and Superior/Inferior) from the end-exhale tumor position was measured from the generated “respiratory-correlated” 4DCT data set. In addition, the range of the external marker positions from the RPM system was measured during 4DCT scanning. Next, the linear correlation between the external marker position (*M*) and the target position (*T*) was obtained as *T* = *αM*, where α is a proportional constant between *T* and *M*. This relationship was subsequently used to calculate the target positions from the abdominal marker positions that were recorded during the beam delivery.

### Treatment planning

Treatment planning was performed on the CT images of the end-exhale phase (typically 50% or 60%) using an Eclipse treatment planning system (Varian Medical Systems). Gross tumor volume (GTV) and surrounding organs were delineated by clinicians. The patients were treated with phase-based gated radiotherapy, which delivers the beam in particular respiratory phases, usually from 30% to 70%. The GTV motion in the gated phases was measured for each direction, and then, the ITV was determined by adding the motion range in each direction anisotropically to the GTV. Planning target volume (PTV) was determined by adding 5-mm of setup margin to the ITV.

All the treatment plans were SBRT using VMAT consisting of two arcs. For 10 lung cancer cases, the prescription dose was 48–60 Gy in 4–5 fractions. The prescription dose to liver patients was 45 Gy in three fractions. The pancreas patients were treated with 26–28 Gy in four fractions. The static 3D dose was calculated using the anisotropic analytical algorithm (AAA) (Varian Medical Systems) with a 2.5-mm grid size on the reference CT image of the end-exhale phase.

### Treatment delivery

All of the 30 patients were treated by respiratory-gated VMAT using a TrueBeam radiotherapy system (Varian Medical Systems) with an HD120 multileaf collimator. The respiratory gated (in general 30–70% of the respiratory phase) VMAT was performed using the RPM system while the patient was breathing freely. For each fraction, kilovoltage (kV) image guidance was performed by the following three steps. First, after the patient setup using the laser system in the treatment room, a set of anterior-posterior and lateral X-ray images was acquired using an on-board imager (OBI) (Varian Medical Systems), and patient alignment based on vertebral bony anatomy was performed. Second, a cone-beam CT (CBCT) was acquired and registered with the plan (end-exhale phase) CT data based on landmarks including tumor, lipiodols, diaphragm, and fiducial markers. The registration was performed considering that the CBCT was acquired slowly over more than one minute, and thus represents motion averaged, while the reference image represents the end-exhale phase CT. Third, gated fluoroscopy in both the AP and lateral direction was monitored. The gated fluoroscopy continuously shows the tumor/surrogates on X-ray fluoroscopy imaging overlaid with the contours of tumor/surrogates from the reference plan CT. With the gated fluoroscopy, it was checked whether the end-exhale position of the tumor/surrogates was the same as the reference position of the end-exhale plan CT. If they were not the same, then couch readjustment was performed for registration. The total number of the individual treatment fractions was 112 (42 from 10 lung patients, 30 from 10 liver patients, and 40 from 10 pancreas patients). For each fraction, two-arc VMAT beams were delivered using the phase-gating technique while the patient was free-breathing. In general, the entire treatment procedure from the patient setup to the completion of beam delivery took 20–30 minutes.

### 4D dose reconstruction

**Fig 1:**
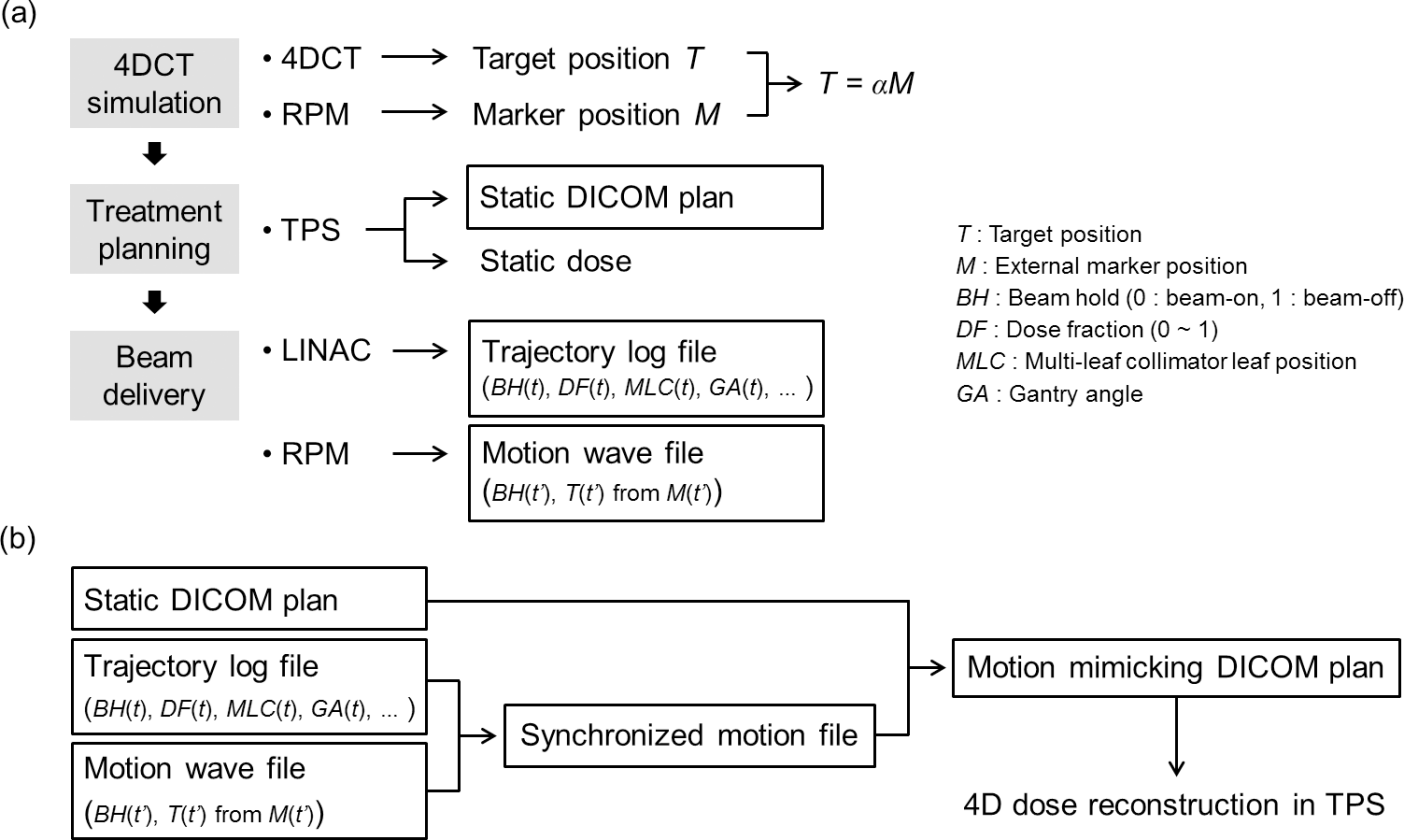
Overview of the 4D dose reconstruction process throughout the entire treatment process. (a) Obtaining data from treatment systems for each process of treatment. (b) Overview of offline data processing for 4D dose reconstruction.

Fig 1 summarizes an overview of the 4D dose reconstruction process along with the necessary data throughout the entire treatment process.

In the 4DCT treatment simulation process, the tumor position as a function of the respiratory phase was measured from the respiratory-phase-sorted 10-bin 4DCT dataset to determine the ITV. A linear correlation of the tumor position and the external marker position (RPM signal), i.e., *T* = *αM*, was calculated and used later for the estimation of the tumor position from the external marker position during treatment beam delivery. Upon completion of treatment planning, the static DICOM plan file was obtained from the Eclipse planning system. The static dose was also calculated with the static plan in the Eclipse planning system.

During each beam delivery, the TrueBeam control system generates a trajectory log file that records the actual machine parameters at 20-ms intervals together with their expected values. It includes the expected and actual values of the gantry angle, collimator angle, jaws positions, couch position, delivered dose in MUs, beam hold status (gated beam on/off), control points, carriage position, and MLC leaf positions. The external marker position of the RPM system is also recorded at 33-ms intervals with beam hold status during the treatment session. The external marker position monitored by the RPM system during treatment was exported from an ARIA oncology information system (Varian Medical Systems) in DICOM waveform format, and which is termed a *motion wave file*. Note that the target position *T*(*t’*) was determined from the marker position *M*(*t’*) of the motion wave file using the linear correlation *T* = *αM*, which was determined during 4DCT simulation as previously mentioned.

Since the motion wave file was acquired from a different computer system than the trajectory log file, the target motion and the treatment machine parameters needed to be synchronized. It was also noted that the time interval of RPM data recorded in the motion wave file had significant variance, which might have occurred due to inaccurate sampling of the computer clock. To solve this issue, each beam-on section in an RPM data point was mapped to the corresponding beam-on section in the trajectory log file, and the time point of the RPM motion wave data was corrected to that of the trajectory log file, which is described as follows.

**Fig 2:**
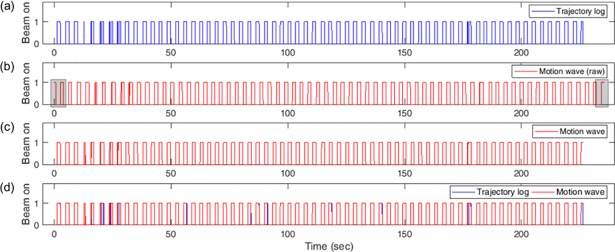
Example of beam hold data for a lung cancer patient; 1 is beam-on and 0 is beam-off. (a) Data of trajectory log file. (b) Original raw data of the motion wave file. The parts to be truncated are indicated as the gray areas. (c) Data of the motion wave file after data processing, including truncation, rescaling, and resampling. (d) Synchronized data for two beam hold data types.

Data processing to synchronize the two different motion data types includes data truncation, rescaling, and resampling in the time dimension. For typical gated radiotherapy, the RPM system is operated for a longer period than for treatment beam delivery. Therefore, motion wave files are longer than trajectory log files, and there are more beam-on sections in motion wave files, as shown in Fig 2(A) and 2(B). Thus, the extra data in the additional beam-on section of the motion wave file had to be truncated. The number of truncated beam-on sections at each end of the motion wave file is determined by minimizing the error rate, which is defined as the percentage of different data points in the truncated beam hold data of the motion wave file compared with the beam hold data of the trajectory log file. Several truncations with different truncation numbers were created, and the truncated data with the lowest error rate was chosen. Before calculating the error rate, the truncated motion wave data were rescaled in order to match the length of the truncated motion wave file to that of the trajectory log file. Further, the truncated motion wave data was resampled into 20-ms time intervals to match those of the trajectory log data. The resulting beam-on data after truncation, rescaling, and resampling is shown in Fig 2(C). Finally, the beam-on sections of the motion wave file were one-to-one matched to the beam-on sections of the trajectory log file to synchronize the exact marker positions in the beam-on sections of the trajectory log file, as shown in Fig 2(D).

**Fig 3:**
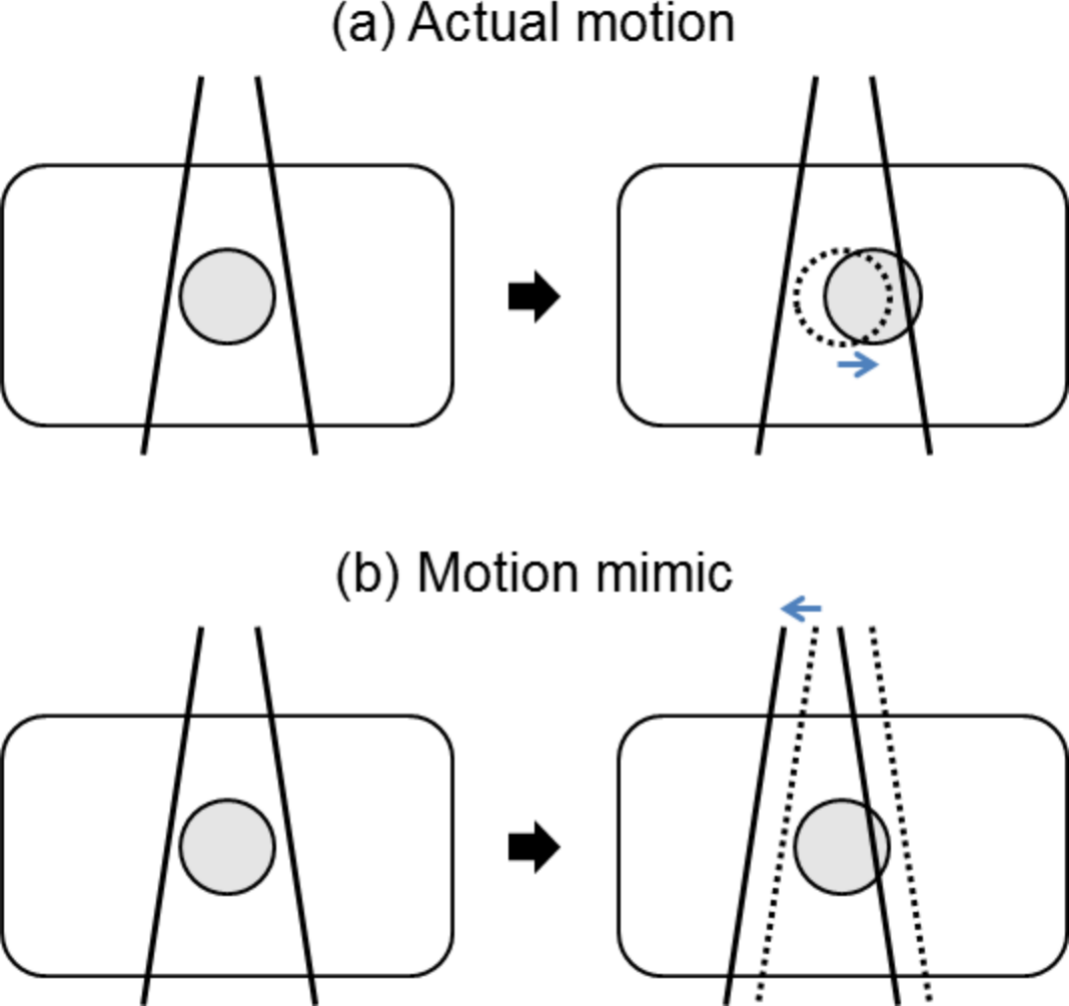
Motion mimicking plan implemented by beam isocenter shifts. If the actual target shifts as indicated by the blue arrow in (a), motion mimicking is implemented by shifting the beam isocenter of the sub-beam in the treatment plan to the opposite direction, as in (b).

To mimic tumor motion in the treatment plan, the beam isocenter was shifted opposite to the tumor movement from the reference (end-exhale) position, as shown in Fig 3. Here, we assume rigid motion for the whole patient that might be reasonable for such a small SBRT tumor, which is generally 2–5 cm in diameter.

It would be ideal to have sub-beams corresponding to every tumor position acquired during treatment. However, this is inefficient and requires time-consuming calculations. Therefore, the tumor motion data was divided into 3D position bins of 1-mm intervals. The same number of sub-beams was formed as the number of divided position bins. Each sub-beam corresponds to a certain position bin with the beam isocenter shifted from the original isocenter as average tumor displacements from the reference position in that position bin.

For details regarding the construction of a sub-beam and how to allocate appropriate machine parameters, refer to Poulsen et al. [23].

For reconstruction of the delivered dose, an in-house program was implemented using MATLAB (R2012a, MathWorks, Natick, MA). The program combined the static treatment plan and motion data, and generated a “motion-mimicking” treatment plan in DICOM-RT format. The motion mimicking plan file was subsequently imported into the Eclipse planning system, and 4D doses were calculated in the planning system.

### Dosimetric analysis

Four-dimensional delivered doses were calculated for 112 treatment fractions and were summed for the total accumulated dose for each of 30 patients. To evaluate the difference between the 4D delivered dose and the static planned dose to GTV and organs at risk (OARs), the maximum dose (D_max_) and mean dose (D_mean_) were calculated. For the lung cancer cases, the OARs included the spinal cord, esophagus, and heart. Liver, duodenum, kidney, spinal cord, and stomach are the OARs considered for the liver and pancreas cancer cases. Minimum dose (D_min_) to GTV was also calculated to verify cold spots in tumors. A two-sided Wilcoxon signed-rank test was performed to test the difference between the dosimetric parameters of the delivered dose distribution and the planned dose distribution. Gamma analysis was performed using OmniPro I’mRT+ software (IBA Dosimetry, Schwarzenbruck, Germany) to compare the 2D dose distributions in the sagittal plane where the planning isocenter is located. The gamma pass criteria was 3% dose difference and 3 mm distance. The percentage dose was calculated relative to the prescription dose, and a dose less than 10% was ignored. The resolution of the dose plane was linearly interpolated to 0.5 mm from the original 2.5-mm grid size of dose calculations in the TPS.

## Results

### Respiratory motion during treatment delivery

Patient characteristics are listed in Table 1.

**Table 1:** Patient characteristics.

		Lung cancer		Liver cancer		Pancreatic cancer	
Age (years)	Median (range)		61		60.5		62
Sex	Male		4 (40%)		9 (90%)		6 (60%)
	Female		6 (60%)		1 (10%)		4 (40%)
ECOG PS	0		4 (40%)		8 (80%)		2 (20%)
	1		3 (30%)		2 (20%)		8 (80%)
	2		3 (30%)		0 (0%)		0 (0%)
Histology		Primary	1 (10%)	HCC	10 (100%)	Adenocarcinoma	10 (100%)
		Metastatic	9 (90%)				
Location		RUL	1 (10%)	Right lobe	7 (70%)	Head	5 (50%)
		RML	1 (10%)	Left lobe	3 (30%)	Body/tail	5 (50%)
		RLL	6 (60%)				
		LLL	2 (20%)				
Stage	I		0 (0%)		5 (50%)		0 (0%)
	II		0 (0%)		4 (40%)		0 (0%)
	III		0 (0%)		0 (0%)		9 (90%)
	IV		10 (100%)		1 (10%)		1 (10%)
Tumor size (cm)	Median (range)		1.3 (0.7–4.2)		1.6 (0.8–4.3)		3.5 (2.0–6.7)
SBRT dose (Gy)	Median (range)		60 (48–60)		45 (35–45)		28 (26–28)
CP class				A	10 (100%)		

ECOG PS, Eastern Cooperative Oncology Group performance status; HCC, hepatocellular carcinoma; RUL, right upper lobe; RML, right middle lobe; RLL, right lower lobe; LLL, left lower lobe; AJCC, American Joint Committee on Cancer; SBRT, stereotactic body radiation therapy; CP, Child-Pugh.

The average tumor motion ranges measured from the 4DCT data were 14.2 mm for the lung, 22.4 mm for the liver, and 9.8 mm for the pancreas, in the superior-inferior (SI) direction, respectively. When applying phase gated (30%–70%) motion management, the motion ranges were reduced to 5.7 mm for the lung, 7.1 mm for the liver, and 4.2 mm for the pancreas in 4DCT data.

Similarly, the tumor motion during the treatment beam delivery was estimated from the RPM signal using the linear correlation (*T* = *αM*) between the external marker and the tumor. To characterize tumor motion measured over multiple cycles during beam delivery, unlike the single respiratory cycle of 4DCT simulation, two types of motion ranges—local and global—were examined in this study. The local motion range is defined as the distance between neighboring inhale and exhale positions. The local motion ranges in the SI direction were 11.3 mm, 14.8 mm, and 8.5 mm, averaged over the 42 lung, 30 liver, and 40 pancreas fractions. For the gated beam-on region, the average gated local motion ranges in the SI direction were 2.6 mm, 3.9 mm, and 2.0 mm for the lung, liver, and pancreas fractions. On the other hand, the global motion range is defined as the distance between the maximum inhale position and minimum exhale position in the total target trajectory of one treatment fraction. Compared with the local range, the global motion range includes the effect of eventual respiratory baseline drift or shift, which results in more significant systematic error in dose distribution. The average global motion ranges in the SI direction were 21.3 mm for the lung, 32.2 mm for the liver, and 18.1 mm for the pancreas fractions. The average gated global motion ranges in the SI direction were 9.6 mm, 11.8 mm, and 6.5 mm for the lung, the liver, and the pancreas fractions, respectively.

**Fig 4:**
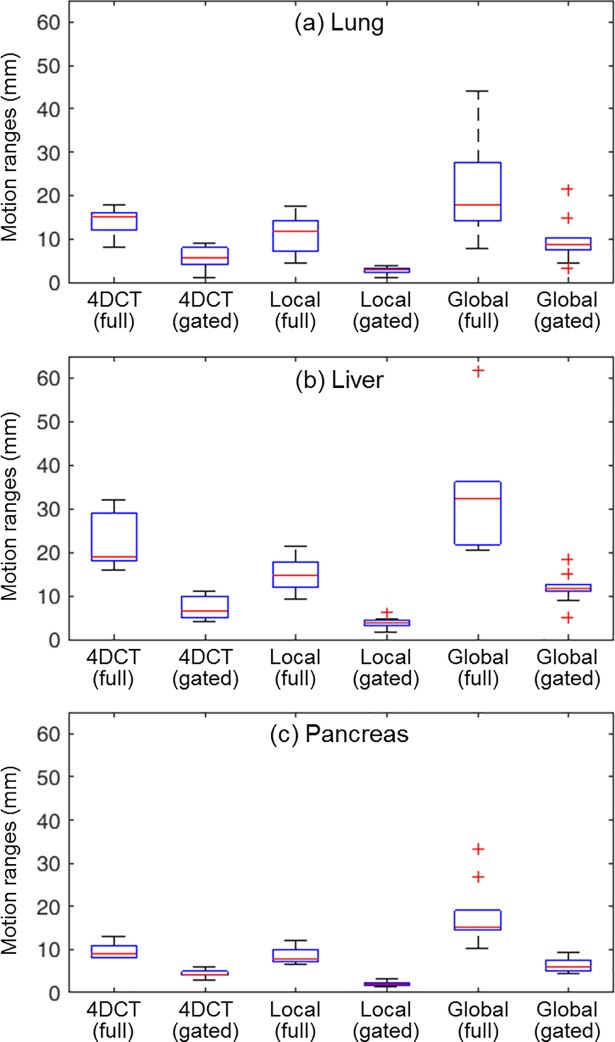
**Motion ranges from 4DCT data before treatment, and local and global motion ranges from the RPM system during treatment for (a) lung, (b) liver, and (c) pancreas patients**. Local and global motion ranges of one patient are averaged for the treatment fractions. The red horizontal line in the boxes represents average motion ranges over 10 patients. The upper line and lower line of a box indicates the 75th percentile and 25th percentile. The whiskers cover ranges in ±2.7σ or 99.3% of the normal distribution. The outliers are indicated as red plus signs.

Fig 4 shows the relationship of the motion ranges from the 4DCT data before treatment and from the RPM system during treatment. The local motion ranges in the actual beam delivery decreased slightly compared with the motion ranges measured in the 4DCT simulation. This can be explained by the fact that, in general, patients became accustomed to the treatment as they were treated in more fractions, and thus their breathing stability was improved after 4DCT treatment simulation. However, the global motion ranges tended to increase compared with the 4DCT motion ranges. This may be because of baseline shifts in breathing during treatment, as increasing time of the treatment procedure. The effect of baseline shifts to motion range and dose distribution change will be discussed in detail.

### 4D delivered dose analysis

Tables 2–4 summarize the changes in dosimetric parameters of GTV and OARs for the patients in the lung, liver, and pancreas cases. The dosimetric parameters, including D_max_, D_mean_, and D_min_, are calculated as the percentage difference of the 4D delivered dose compared with the 3D planned dose. No significant difference was observed in the GTV dose parameters. For the lung cases, the D_max_ of right lung and the D_mean_ of left lung showed small increases of 0.5% and 0.9%, respectively, but clinical impact would be little. For the liver cases, the difference of D_max_ and D_mean_ in the duodenum, stomach, and left and right kidney showed statistical significance. For those OARs, the delivered doses were all decreased compare with the planned doses. This is expected because the organs below the tumor in the SI direction are likely to be underdosed compared with the static plan based on the end-exhale phase of respiratory motion. In contrast, the organs above the target are like to be overdosed. However, the difference was not clinically significant, and ranged from 3.1% to 4.2%, as shown in Table 3. For the pancreas cases in Table 4, the difference of the D_mean_ of the liver, duodenum, stomach, and right kidney, and the difference of the D_max_ were statistically meaningful. The mean dose decreased slightly in the duodenum and right kidney, both of which are located under the tumor, while it increased in the liver and stomach, both of which are located above the tumor. Furthermore, the magnitude of the percent differences was approximately 1–2%, which is smaller than for the liver cases. This smaller dose difference for the pancreas seems reasonable considering smaller motion ranges compared with the other cases.

**Table 2:** The dosimetric parameters for the 10 lung cases.

	Percentage of dose difference (normalized 100% to the prescription dose)					
	GTV	Esophagus	Heart	Spinal cord	Left lung	Right lung
D_max_	0.3 ± 0.7 (0.322)	-0.8 ± 2.1 (0.492)	0.0 ± 0.9 (0.625)	-0.6 ± 2.6 (0.846)	-0.8 ± 1.2 (0.275)	0.5 ± 0.9 (0.049)
D_mean_	0.0 ± 0.7 (0.789)	-0.3 ± 0.8 (0.133)	0.9 ± 2.3 (0.275)	-0.1 ± 1.4 (0.484)	0.9 ± 0.9 (0.002)	0.7 ± 1.9 (0.375)
D_min_	0.0 ± 0.5 (1)					

Percentage of dose difference (normalized 100% to the prescription dose) between the 4D delivered dose and the static planned dose in average ± standard deviation (p-value).

**Table 3:** The dosimetric parameters for the 10 liver cases.

	Percentage of dose difference (normalized 100% to the prescription dose)						
	GTV	Liver	Duodenum	Stomach	Spinal cord	Left kidney	Right kidney
D_max_	0.1 ± 0.9 (0. 922)	-0.1 ± 1.0 (0.537)	-4.2 ± 1.4 (0.002)	-3.5 ± 4.2 (0.020)	-0.6 ± 1.3 (0.432)	-4.1 ± 2.8 (0.002)	-3.2 ± 1.3 (0.002)
D_mean_	0.2 ± 0.6 (0.695)	-1.2 ± 2.3 (0.193)	-3.8 ± 1.4 (0.002)	-3.9 ± 2.2 (0.002)	0.4 ± 0.9 (0.346)	-3.1 ± 2.8 (0.016)	-4.1 ± 2.6 (0.004)
D_min_	0.1 ± 1.7 (0.232)						

Percentage of dose difference (normalized 100% to the prescription dose) between the 4D delivered dose and the static planned dose in average ± standard deviation (p-value).

**Table 4:** The dosimetric parameters for the 10 pancreas cases.

	Percentage of dose difference (normalized 100% to the prescription dose)						
	GTV	Liver	Duodenum	Stomach	Spinal cord	Left kidney	Right kidney
D_max_	0.1 ± 0.4 (0.846)	1.6 ± 4.9 (0.375)	-0.9 ± 2.3 (0.375)	2.1 ± 3.0 (0.014)	1.9 ± 4.3 (0.375)	-0.6 ± 1.7 (0.557)	0.0 ± 1.8 (1)
D_mean_	-0.1 ± 0.1 (0.064)	1.5 ± 0.6 (0.002)	-1.0 ± 1.4 (0.049)	2.1 ± 1.6 (0.002)	0.2 ± 0.4 (0.434)	-0.4 ± 0.9 (0.422)	-1.3 ± 0.9 (0.008)
D_min_	-1.6 ± 2.6 (0.064)						

Percentage of dose difference (normalized 100% to the prescription dose) between the 4D delivered dose and the static planned dose in average ± standard deviation (p-value).

**Fig 5:**
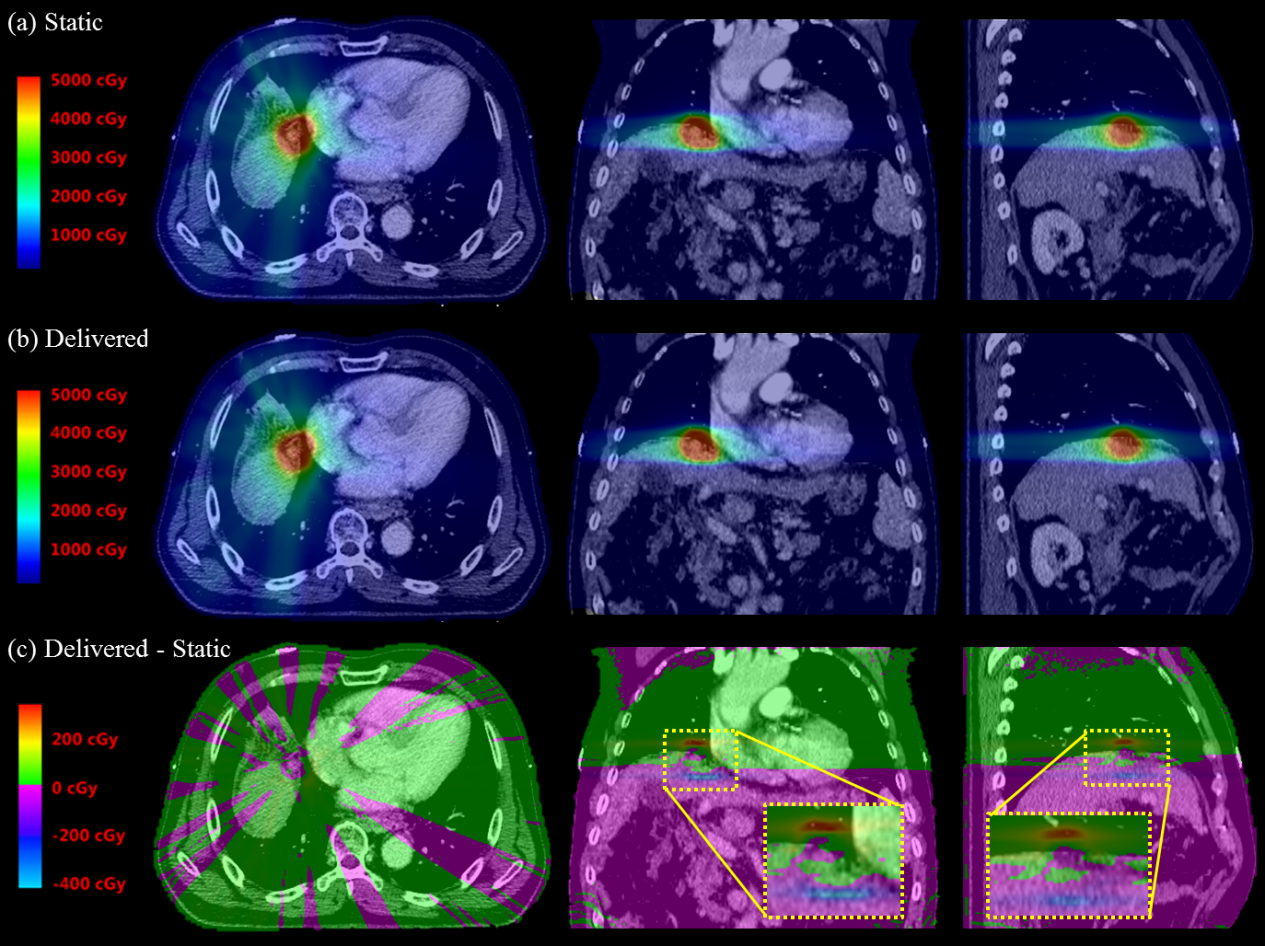
An example of dose distribution changes for a liver case. The prescription dose was 45 Gy in three fractions. The static dose distribution in the original plan is shown in (a), and the 4D delivered dose distribution is shown in (b). The difference between the delivered and the static dose distribution is shown in (c).

Fig 5 presents dose distribution changes between the static and delivered plans for a liver case. In the coronal and sagittal views of Fig 5(C), some hot spot dose regions (in red) are found above the tumor, while cold spots (in blue) occurred below the tumor. This is because in actual treatment due to respiratory motion, the overall tumor position is lower than the top position where the tumor would stay at the end-exhale phase on the static plan. While the prescription dose was 45 Gy in three fractions, the maximum increase of the delivered dose to the static planned dose was 3.37 Gy for the hot spots, and the maximum decrease was -4.72 Gy for the cold spots.

### Gamma analysis

**Fig 6:**
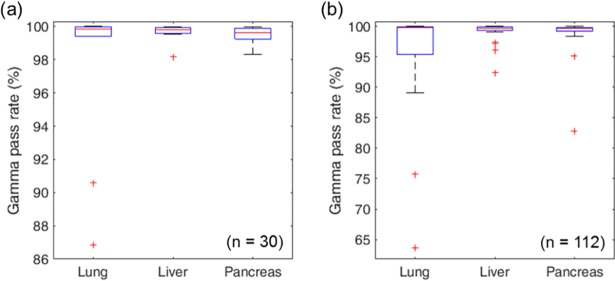
**Box plotted distribution of the gamma pass rates for (a) 30 patients and for (b) 112 treatment fractions.** The upper line and lower line of a box indicates the 75th percentile and 25th percentile. The whiskers cover ranges in ±2.7σ or 99.3% of the normal distribution. The outliers are indicated as red plus signs.

Fig 6 shows the distribution of gamma pass rates (3 mm / 3%) of the delivered dose compared with the static dose for each patient and each fraction. The average gamma pass rates were 97.6 ± 4.8 for lung cases, 99.6 ± 0.5 for liver cases, and 99.5 ± 0.5 for pancreas cases. Most cases of the patients showed insignificant dose variation with gamma pass rates higher than 98% except for two lung cases with gamma pass rates of 86.9% and 90.6%, as shown in Fig 6(A). Meanwhile, the gamma pass rates for individual treatment fractions show worse results than those for patients, indicating that certain fractions had more error than the others. However, the total accumulated dose for each patient is usually in better agreement.

**Fig 7:**
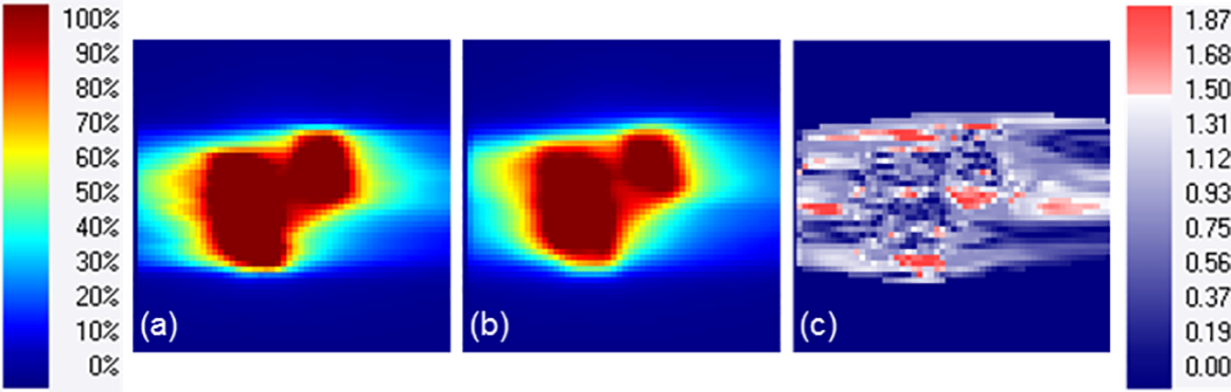
Sagittal distribution of (a) a planned dose, and (b) a 4D delivered dose in percentage of the 60-Gy prescribed dose for the lung case with minimum gamma pass rate of 86.9%. (c) The gamma index map in 3 mm / 3% criteria.

Fig 7 shows an example of gamma evaluation of a lung patient with the minimum gamma pass rate of 86.9% among a total of 30 patients. The gated SI global motion range of this patient was an average of 21.7 mm for all the treatment fractions.

**Fig 8:**
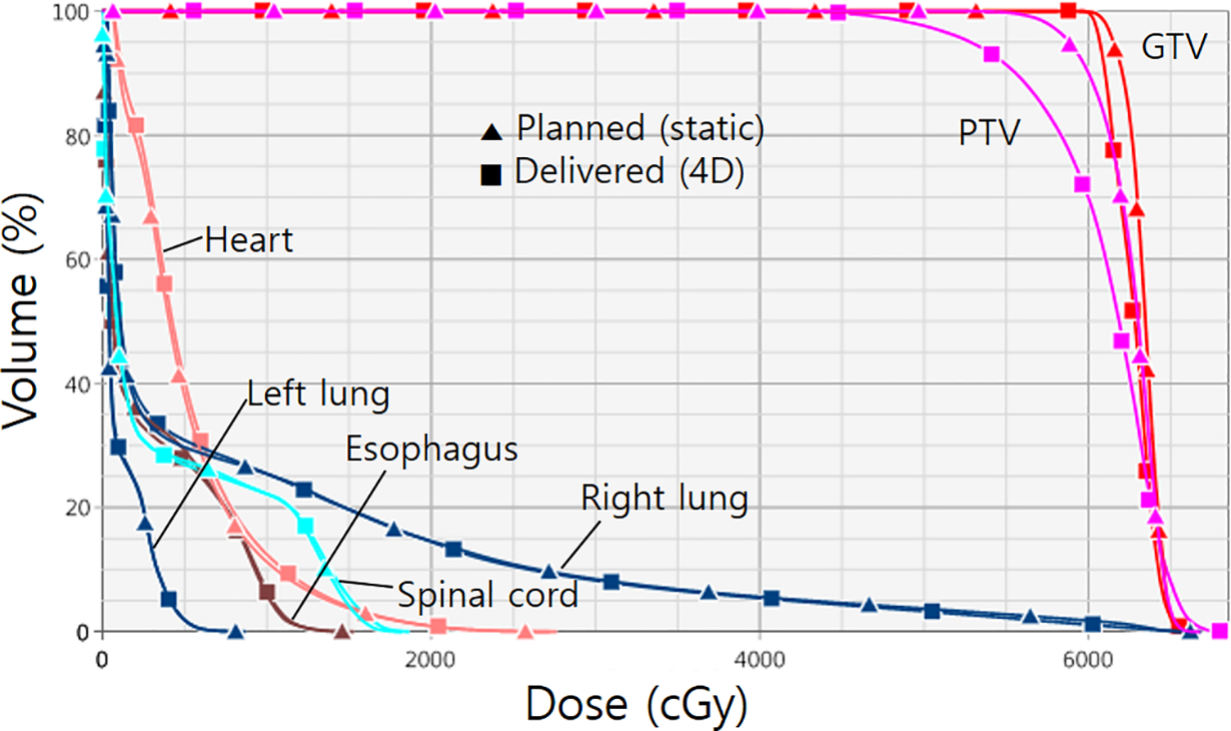
An example of the dose-volume histogram (DVH) showing the differences between the delivered and planned dose.

Fig 8 illustrates changes in the dose-volume histogram (DVH) between the delivered dose and the planned dose for the same patient, as shown in Fig 7. The DVH of the PTV is significantly deteriorated by respiratory motion with a baseline shift in the delivered dose. The steep DVH line of the GTV in the static plan is significantly deteriorated by respiratory motion with baseline shift in the delivered dose, but the minimum coverage of GTV is still maintained with the prescription dose of 60 Gy. This is because the PTV margin of 5 mm can effectively cover the positional variation of GTV due to the baseline shift. For the OARs, there is no significant dosimetric difference, except that the ipsilateral right lung dose around 500 cGy was slightly increased in the delivered dose as the tumor volume for the SBRT was relatively small.

This patient received SBRT of 60 Gy for a lung metastasis from rectal cancer. He gained complete response of the tumor at four months after SBRT, and there was no evidence of tumor recurrence until the last follow-up (27 months after SBRT). Grade 1 focal radiation fibrosis was found at eight months after SBRT, although without any related symptoms.

## Discussion

Comparison of the dose distribution between the static planned dose and the delivered dose under breathing motion for 30 gated VMAT SBRT patients was performed using 4D dose calculation with a motion mimicking plan in a clinical TPS. Three groups of tumor sites, including lung, liver, and pancreas were evaluated. Discrepancy of the dose distribution between the planned and delivered doses was evaluated using gamma analysis. Most of the patients showed gamma pass rates over 98%, and thus confirming that the static plan dose with the end-exhale CT can represent the actual delivered dose, at least in the case of the gated VMAT of this study, although breathing motion was involved.

One of the main observations of the results was no statistical difference in the GTV dosimetric parameters. Since any statistically significant difference could be missed due to the small sample size of the study (N = 10), we performed a power analysis using the PASS 11 software (NCSS Statistical Software, Kaysville, UT, USA). Statistical powers as a function of paired differences for one of the dosimetric parameters (D_max_) between the planned and delivered dose are shown in S1 Fig. A sample size of 10 achieves 80% power to detect a mean of paired differences of 0.9 with an estimated standard deviation of differences of 0.9 (maximum SD in liver cases) and with a significance level (alpha) of 0.05 using a two-sided Wilcoxon test, assuming that the actual distribution is uniform. We think that a difference of 0.9, which means the % difference from the prescription dose, is not a clinically meaningful difference. Therefore, we can conclude that the sample size of 10 has enough statistical power to detect the clinically relevant dose difference for the GTV, assuming that the dose difference smaller than 0.9% of the prescription dose is not clinically relevant.

Several studies have evaluated accumulated dose differences caused by respiratory-induced organ motion and deformation using 4D dose calculations with deformable image registration (DIR) of 4DCT images [18]^,^[19]. These studies suggest that a 4D plan considering respiratory-induced organ motion and deformation could improve the accuracy of an accumulated dose, although, in general, the difference is not significant. However, these simulation studies of 4DCT are reflecting real situation in a limited way by assuming that breathing motion would be the same all the time from 4DCT to every fraction of treatment. In reality, patient breathing patterns during beam delivery can change from that of the 4DCT simulation. Baseline shift, irregular respiratory amplitude, and a phase difference in respiratory motion can occur during beam delivery and could cause a significant dose difference from the expected plan dose. Likewise, the studies on the interplay effect were also performed by simulating target motion using sinusoidal phantom motion [27], 4DCT data [28], and motion of the treatment machine, including multileaf collimator (MLC) leaf motion, jaw movement, gantry rotation, etc. These studies concluded that the interplay effect on the accumulated dose difference is not significant. However, the simulation studies using sinusoidal or breathing patterns from 4DCT data may not be accurate enough because accurate reflection of the interplay effect needs the trajectories of dynamic machine parameters and target motion actually delivered during beam delivery.

**Fig 9:**
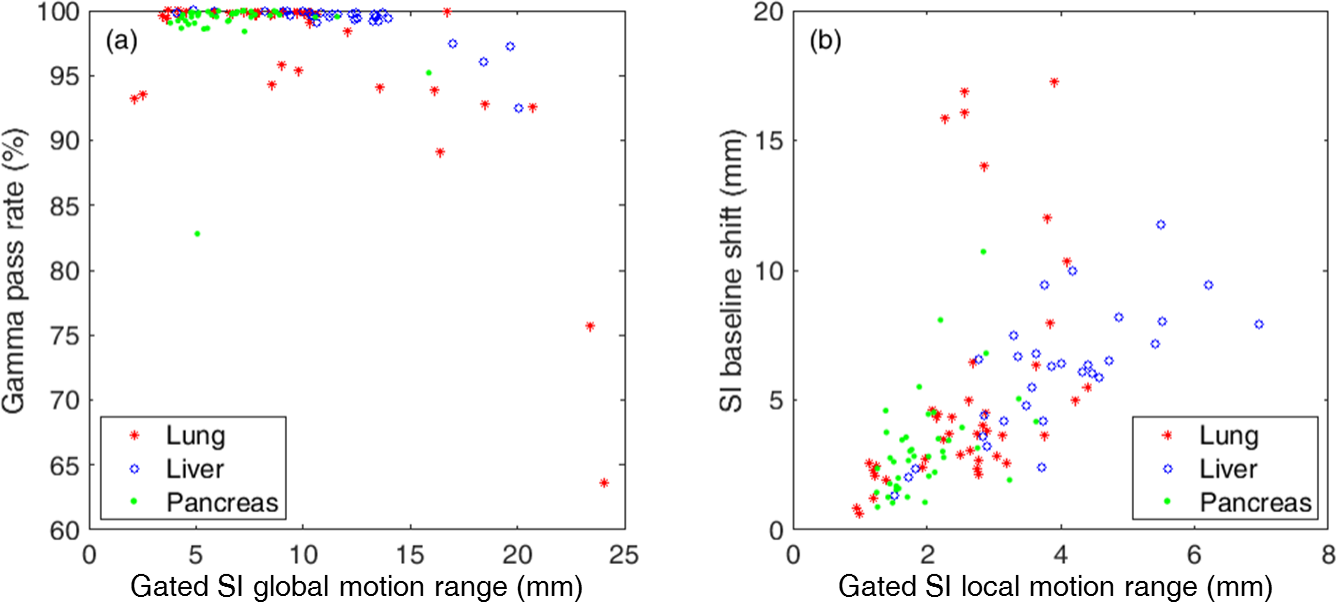
**(a) Gated SI global motion range of the targets versus gamma pass rate for each fraction of the treatments. (b) Gated local motion range versus baseline shift for the SI direction.** Total numbers of treatment fractions are 42 for the lung, 30 for the liver, and 40 for the pancreas treatments.

Fig 9(A) shows the effect of the global motion range to the gamma pass rates. The dose discrepancy does not seem to be directly proportional to the gated global motion range until 15 mm, then tends to decrease quickly, especially for lung cases. In addition, the gamma pass rates of lung cases are slightly lower than for the liver/pancreas cases. It is thought that the rapid density changes between a solid lung tumor and the surrounding lung tissue creates more sensitivity to the dose distribution changes caused by motion, unlike the liver and pancreas cases where surrounding organs have similar density.

Furthermore, the global motion range could increase when either the local motion ranges or the baseline shift increases. Hence, the baseline shift was measured by the difference between the maximum position and the minimum position at the end-exhale phase for the target trajectories from one treatment fraction. Fig 9(B) shows increases in the baseline shift as the local motion range increases with some exceptions of lung cases.

**Fig 10:**
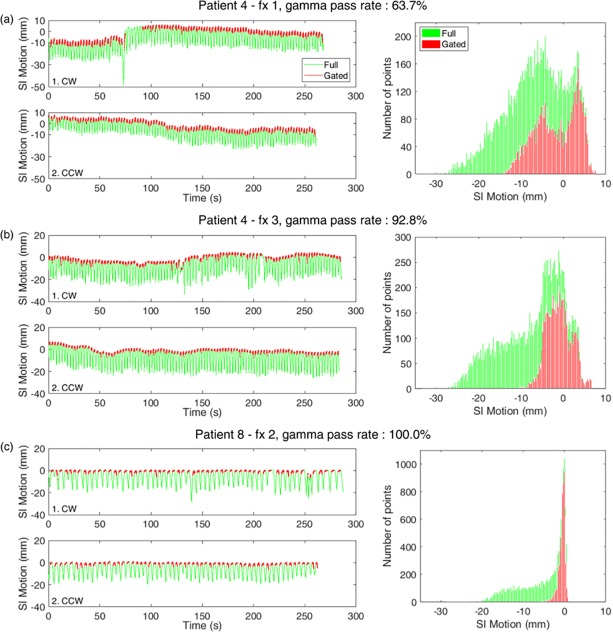
**Examples of lung tumor trajectories of full respiratory phases (green) and beam-on positions in gated phases (red) from three SBRT fractions using two VMAT beams (1. CW, and 2. CCW) on the right lower lobe of (a) lung patient no. 4 with lowest gamma pass rate, (b) relatively lower than other patients but highest among the fractions of patient no. 4 and (c) patient no. 8 with the highest gamma pass rate among all lung cases.** For each case, the histogram of the SI motion distribution for full (green) and gated (red) phases is shown on the right side for each SBRT fraction.

An example of the lowest dose discrepancy case, which has a 86.9% gamma pass rate for total treatment, was a lung cancer patient (patient no. 4), shown in Fig 10. The prescribed dose was 60 Gy in four fractions for the treatment. Among the four treatment fractions, the lowest gamma pass rate was 63.7% for the first fraction (fx 1), and the highest was 92.8% for the third fraction (fx 3). The actual motion during the treatment delivery for each fraction is shown in Fig 10(A) for fx 1, and in Fig 10(B) for fx 3. Target SI motions as a function of time are displayed, while distributions of SI position during treatment fraction are presented on the right side. Fig 10(C) is shown for comparison as the lowest level of dose discrepancy, which has a 100.0% gamma pass rate (patient no. 8, fx 2).

Both patients no. 4 and no. 8 were treated for tumors in the right lower lobe of the lungs. For the worst case, with the gamma pass rate of 63.7%, the target motion shifted once rapidly in the clockwise (CW) arc field of the VMAT and gradually shifted down in the counterclockwise (CCW) arc field, as shown in Fig 10(A). A sudden drift of the baseline resulted in two peaks in the histogram. The second case of fx 3 of patient no. 4 in Fig 10(B) shows a certain baseline shift, but one that is smaller than the first fraction. The position distribution in the histogram is more focused compared with the distribution of fx 1. The lesson we learned from this case was that phase gating can be significantly inaccurate if the baseline shift is not checked. In comparison with patient no. 4, patient no. 8 showed a stable breathing pattern, as shown in Fig 10(C), which is suitable for the phase gating. The gated position is well focused to the treatment isocenter.

One of the limitations of this study is that we did not directly measure the target position. Instead, a simple linear correlation was used to determine the target positions from the external marker positions from RPM. Although more elaborate respiratory model was suggested by Zhang et al.[29], an accuracy of liner motion correlation model between the motion of an external marker and an internal marker has been estimated as 1 mm or less [26]. This error caused by the linear correlation model is small enough to ignore, considering that the grid size of the dose calculation was 2.5 mm.

Another limitation is that the in-house program only handles Varian trajectory log files, but since Elekta also provides similar machine records (TRF log files), the files can be easily adapted.

Furthermore, note that the method of the 4D dose reconstruction used in this study is only considered for target motion, machine motion, and the interplay effect between them. However, there could be anatomic deformation of the target and normal organs in actual breathing. There could be also a residual setup error even after tumor registration using on-line CBCT. As previously mentioned, the registration was performed considering that the CBCT was acquired slowly over more than one minute, and thus represents motion averaged, while the reference image represents the end-exhale phase CT. Recently a gated CBCT [30] is also available, and thus potentially reduce the setup error.

Nevertheless, the proposed method that can assess the actual delivered dose is successfully demonstrated to be quite useful for QA purposes of respiratory motion management because uncontrolled situations such as baseline shift, irregular respiratory amplitude, and phase difference in respiratory motion may not be accounted for during the planning stage. Therefore, 4D dose reconstruction is required for more accurate evaluation of the dose actually delivered.

## Conclusions

The actual delivered dose in thoracic and abdominal VMAT under breathing motion was verified by 4D dose reconstruction using standard treatment equipment and software. The dosimetric change induced by patient and machine motion effects was not significant overall. However, a few treatments, especially for lung cases, showed potentially significant changes because of unexpected changes of patient motion in actual beam delivery from treatment simulation and planning. This study showed a useful verification method of actual delivered dose distribution and provided an understanding of the relationship between breathing motion and dosimetric change at different tumor sites. The method used in this study could be applied for a wide range of cases of dosimetric verification for radiation treatment under various respiratory management techniques, such as gating and tracking. This study also demonstrated that the method can be a useful QA tool for delivered dose verification for gated radiotherapy.

## Supporting information

S1 Fig Statistical powers as a function of paired differences for a dosimetric parameter (D_max_) between the planned and delivered dose by each cancer, which was calculated using the PASS software. (TIF)
