# Supplementary figures and images for: Evaluation of delivered dose to a moving target by 4D dose reconstruction in gated volumetric modulated arc therapy
Source: PLoS One. 2018 Sep 7;13(9):e0202765. doi: 10.1371/journal.pone.0202765 (PMC6128520; doi:10.1371/journal.pone.0202765)

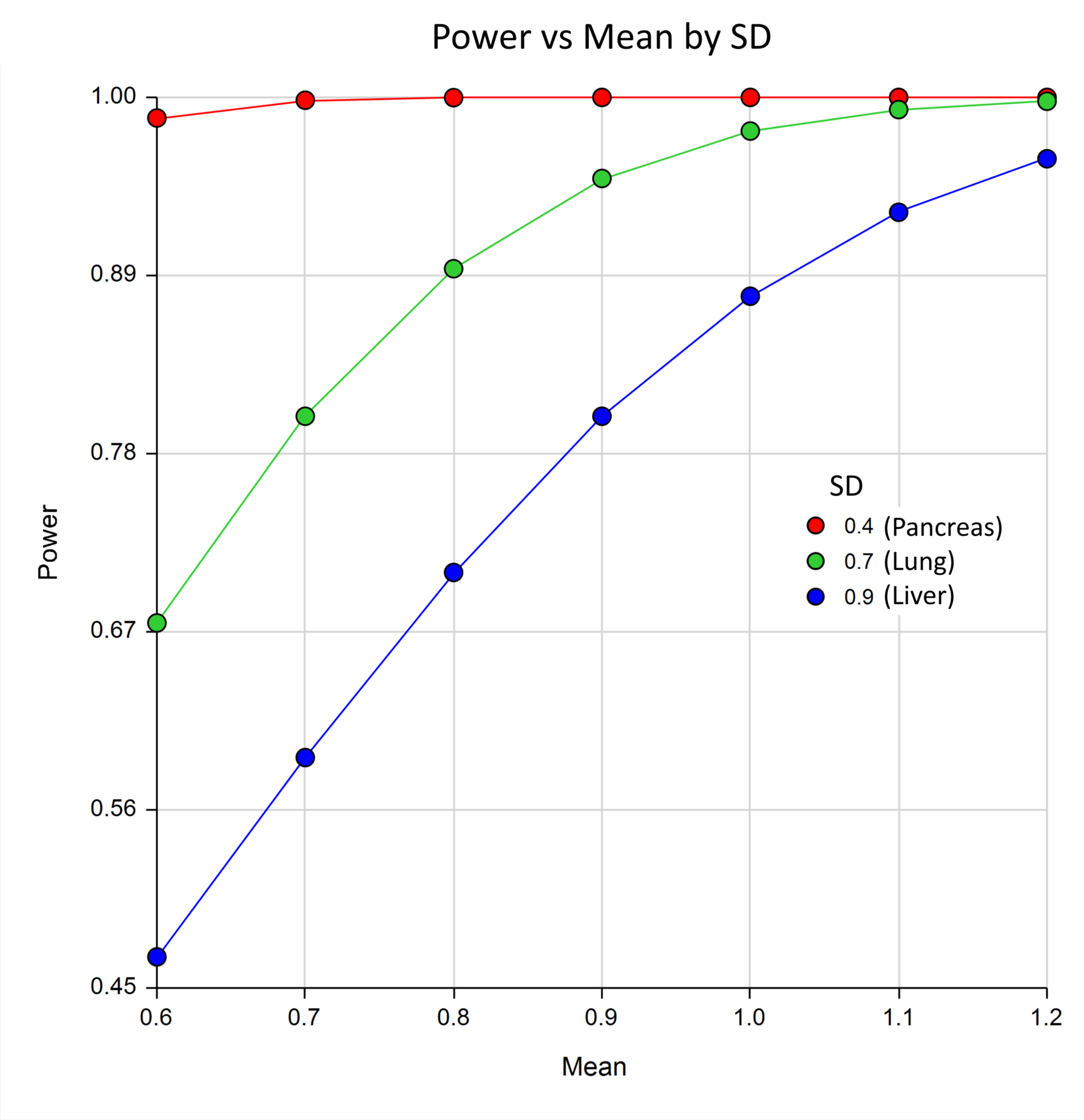

Supplement: S1 Fig — (TIF) [file pone.0202765.s001.tif]
